# Precision of a new ocular biometer in eyes with cataract using swept source optical coherence tomography combined with Placido-disk corneal topography

**DOI:** 10.1038/s41598-017-13800-7

**Published:** 2017-10-23

**Authors:** Wenwen Wang, Yaxin Miao, Giacomo Savini, Colm McAlinden, Hao Chen, Qingjie Hu, Qinmei Wang, Jinhai Huang

**Affiliations:** 1 School of Ophthalmology and Eye Hospital, Wenzhou Medical University, Wenzhou, Zhejiang, China; 2 G.B. Bietti Foundation IRCCS, Rome, Italy; 3 University Hospitals Bristol NHS Foundation Trust, Bristol, UK

## Abstract

The present study was to assess the precision (repeatability and reproducibility) of a new optical biometer (OA-2000, Tomey, Japan) based on swept-source optical coherence tomography (SS-OCT) and Placido disk topography in eyes with cataracts. Seventy-eight eyes from seventy-eight patients with cataracts were evaluated. Axial length (AL), anterior chamber depth (ACD), keratometry (K) over a 2.5 mm and 3.0 mm diameter, lens thickness (LT), central corneal thickness (CCT) and white-to-white (WTW) distance were measured by 2 skilled operators. OA-2000 measurements were highly repeatable and reproducible for all parameters (intraclass correlation, 0.925 to 1.000). OA-2000 derived K-values with a diameter of 3.0mm showed narrower 95% limits of agreement (LoA) (SRK/T: −0.18 to 0.16D; Holladay 1: −0.20 to 0.19D; Hoffer Q: −0.22 to 0.20D) than those with a diameter of 2.5 mm for IOL power calculations (SRK/T: −0.20 to 0.20D; Holladay 1: −0.23 to 0.23D; Hoffer Q: −0.25 to 0.25D). The precision (repeatability and reproducibility) of the OA-2000 was excellent for all parameters. The 3.0mm diameter K-readings appear to be the most reliable choice for calculation of IOL power with the OA-2000. In addition, the average values determined from each operator’s 3 consecutive readings were more reproducible.

## Introduction

Accurate calculation of the intraocular lens (IOL) power is a mandatory step to achieve the desired refractive outcome following cataract surgery. IOL power calculation depends on precise and accurate measurements of the ocular biometric parameters^1–3^. Keratometry (K), i.e. the measurement of corneal curvature and calculation of its power, can be performed by several technologies, whereas axial length (AL) and anterior chamber depth (ACD) are assessed either by ultrasound biometry (preferably with the immersion technique)^4,5^ and optical biometry. Since the introduction of the IOLMaster (Carl Zeiss Meditec, Germany), optical biometry has gradually replaced traditional ultrasound biometry. IOLMaster applies partial coherence interferometry (PCI) for AL measurements, lateral slit illumination for anterior chamber depth (ACD) measurements and automated keratometry with six peripheral measuring points for corneal curvature estimation^6–9^. It is non-contact device and thus does not require topical anesthesia and does not induce any risk of infection. Furthermore, many published studies have indicated that the device shows good repeatability and reproducibility^6,10–13^.

Recently, a newly developed optical biometry device (OA-2000, Tomey, Japan), which combines swept source optical coherence tomography (SS-OCT) and Placido disk topography, has been introduced into the market. The new device can measure seven ocular parameters: AL, ACD, K over a 2.5mm and 3.0mm diameter, lens thickness (LT), pupil diameter (PD), central corneal thickness (CCT) and white-to-white (WTW) distance. We recently demonstrated that the OA-2000 SS-OCT biometer and the IOLMaster PCI biometer provided measurements with high agreement for most biometrical parameters^14^. To our knowledge, however, the precision (repeatability and reproducibility) has not yet been investigated in eyes with cataract, and no studies have assessed whether an average of consecutive readings or a single reading is better. Therefore, the aim of this study was to prospectively evaluate the repeatability and reproducibility of the measurements with this new SS-OCT based biometer in eyes with cataract, test the IOL power calculation obtained with these measurements, and assess whether the average or single measurement is better in clinical practice.

## Results

This prospective study compromised randomly 78 right eyes of 78 patients with cataracts (41 men, 37 women).The mean age of the patients was 68.12 ± 8.82 years (range, 45 to 85 years).

### Intraoperator repeatability for biometry

The OA-2000 provided highly repeatable measurements for AL, CCT, ACD, LT, WTW and K values, with both operators (Table 1). The ICCs of all ocular components were higher than 0.94, and the CoVs were less than 0.76% except LT (less than 2.07%) and ACD (less than 1.43%). Measurement of the AL provided the highest repeatability as the repeatability limit (*r*) was lower than 0.05mm and the ICC between 0.999 and 1.0.

**Table 1:** Intraobserver repeatability outcomes for biometric measurements obtained using OA-2000 swept-source optical coherence tomography in cataract patients.

Parameter	observer	Mean ± SD	Sr	Repeatability limit (*r*)	COV (%)	ICC (95% CI)
AL (mm)	1st	23.36 ± 0.92	0.02	0.05	0.07	1.000 (0.999 to 1.000)
	2nd	23.36 ± 0.92	0.02	0.05	0.08	1.000 (0.999 to 1.000)
CCT (μm)	1st	518.25 ± 33.20	3.94	10.92	0.76	0.986 (0.980 to 0.991)
	2nd	518.82 ± 32.65	3.71	10.29	0.72	0.987 (0.981 to 0.991)
ACD (mm)	1st	3.03 ± 0.33	0.03	0.09	1.12	0.990(0.985 to 0.993)
	2nd	3.03 ± 0.33	0.04	0.12	1.43	0.983 (0.976 to 0.989)
LT (mm)	1st	4.51 ± 0.38	0.09	0.26	2.07	0.943 (0.918 to 0.961)
	2nd	4.51 ± 0.38	0.09	0.24	1.93	0.950 (0.929 to 0.966)
Ks (Φ = 2.5) (D)	1st	44.68 ± 1.57	0.13	0.36	0.29	0.993 (0.990 to 0.995)
	2nd	44.69 ± 1.56	0.12	0.34	0.28	0.994 (0.991 to 0.996)
Kf (Φ = 2.5) (D)	1st	43.95 ± 1.61	0.15	0.41	0.33	0.992 (0.988 to 0.994)
	2nd	43.95 ± 1.61	0.16	0.45	0.37	0.990 (0.985 to 0.993)
Km (Φ = 2.5) (D)	1st	44.32 ± 1.58	0.10	0.29	0.23	0.996 (0.994 to 0.997)
	2nd	44.32 ± 1.57	0.11	0.31	0.25	0.995 (0.993 to 0.997)
Ks (Φ = 3.0) (D)	1st	44.65 ± 1.57	0.12	0.33	0.27	0.994 (0.992 to 0.996)
	2nd	44.64 ± 1.57	0.13	0.35	0.29	0.993 (0.990 to 0.996)
Kf (Φ = 3.0) (D)	1st	43.96 ± 1.63	0.13	0.37	0.31	0.993 (0.990 to 0.995)
	2nd	43.96 ± 1.62	0.15	0.41	0.34	0.992 (0.988 to 0.994)
Km (Φ = 3.0) (D)	1st	44.30 ± 1.58	0.09	0.25	0.21	0.997 (0.995 to 0.998)
	2nd	44.30 ± 1.59	0.11	0.29	0.24	0.993 (0.994 to 0.997)
WTW (mm)	1st	11.50 ± 0.37	0.09	0.24	0.76	0.947 (0.924 to 0.964)
	2nd	11.49 ± 0.35	0.08	0.22	0.70	0.950 (0.929 to 0.966)

AL = Axial length, CCT = central corneal thickness, ACD = anterior chamber depth, LT = lens thickness, K = keratometry, WTW = white to white, SD = standard deviation, Sr = within-subject standard deviation, COV = within-subject coefficient of variation, ICC = intraclass correlation coefficient.

### Interoperator reproducibility for biometry

#### Average of 3 consecutive readings

Table 2 shows the interoperator reproducibility of the average measurements for all parameters obtained by averaging each operator’s 3 consecutive readings. There was no statistically significant difference between the two operators’ mean measurements (*P* > 0.05). All the ICC values were higher than 0.925, demonstrating an excellent reproducibility for all parameters. The Bland-Altman plots showed narrow 95% LoA for the AL, CCT, ACD, K values, and WTW measurements, and a fixed bias was not detected between the two operators. Measurement of the AL provided the highest reproducibility as the ICC between 0.999 and 1.0 and the 95% LoA between −0.02 and 0.03 mm.

**Table 2:** The mean difference, paired t-test, 95% LoA and ICC for biometric measurements differences between the two different operators based on the average method (from average of 3 consecutive readings from each operator) using OA-2000 swept-source optical coherence tomography in cataract patients.

Device Pairings	Mean Difference ± SD	*P* Value*	95% LoA	ICC (95% CI)
AL (mm)	0.00 ± 0.01	0.541	−0.02 to 0.03	1.000 (0.999 to 1.000)
CCT (μm)	−0.58 ± 2.98	0.092	−6.43 to 5.27	0.996 (0.993 to 0.997)
ACD (mm)	0.00 ± 0.03	0.565	−0.06 to 0.06	0.996 (0.993 to 0.997)
LT (mm)	0.00 ± 0.08	0.647	−0.16 to 0.15	0.979 (0.967 to 0.987)
Ks (Φ = 2.5) (D)	−0.01 ± 0.12	0.706	−0.25 to 0.24	0.997 (0.995 to 0.998)
Kf (Φ = 2.5) (D)	0.00 ± 0.13	0.853	−0.25 to 0.25	0.997 (0.995 to 0.998)
Km (Φ = 2.5) (D)	0.00 ± 0.09	0.924	−0.19 to 0.18	0.998 (0.997 to 0.999)
Ks (Φ = 3.0) (D)	0.01 ± 0.13	0.543	−0.24 to 0.26	0.997 (0.995 to 0.998)
Kf (Φ = 3.0) (D)	0.00 ± 0.10	0.892	−0.19 to 0.20	0.998 (0.997 to 0.999)
Km (Φ = 3.0) (D)	0.01 ± 0.08	0.556	−0.15 to 0.16	0.999 (0.998 to 0.999)
WTW (mm)	0.01 ± 0.14	0.542	−0.27 to 0.29	0.925 (0.886 to 0.952)

AL = Axial length, ACD = anterior chamber depth, K = keratometry, WTW = white to white, SD = Standard deviation.

*Two ways.

#### Single reading

**Figure 1:**
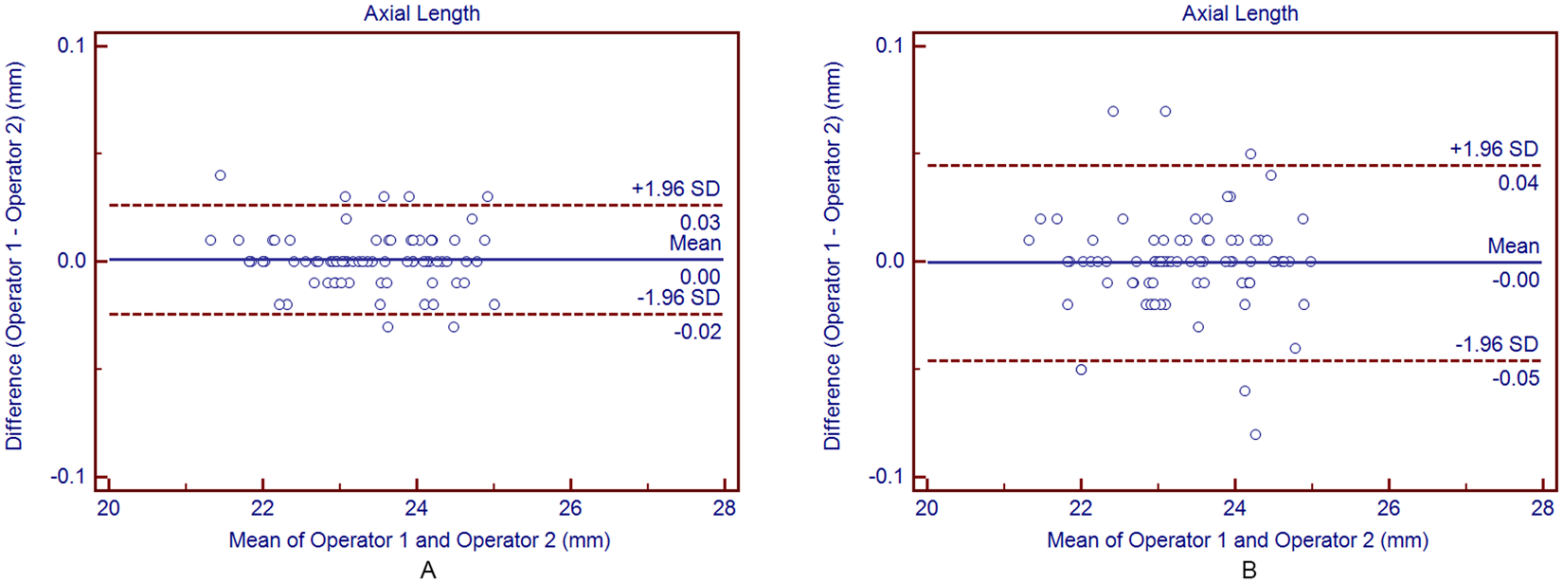
Bland-Altman plots showing agreement in axial length measurements between the 2 operators based on average method (**A**) and on the single method (**B**). The solid line indicates the mean difference (bias). The upper and lower dashed lines represent the 95% LoA.

**Figure 2:**
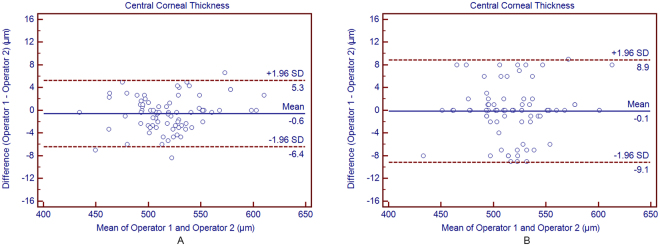
Bland-Altman plots showing agreement in central corneal thickness measurements between the 2 operators based on average method (**A**) and on the single method (**B**). The solid line indicates the mean difference (bias). The upper and lower dashed lines represent the 95% LoA.

Table 3 shows the interoperator reproducibility of all parameters obtained from each operator’s first single OA-2000 reading. There was no statistically significant difference between the two operators (*P* > 0.05). The ICCs for AL, CCT, ACD, K values were more than 0.990, revealing excellent reproducibility. The LT and WTW showed relativity lower but also high reproducibility with ICC were 0.923, 0.897, respectively. The 95% LoA obtained by the first single reading from each operator were statistically wider than those of three consecutive mean readings. The width of the 95% LoA for AL, CCT, LT, Km(2.5), Km(3.0), WTW based on average method were decreased by 44.4%, 35%, 48.3%, 42.2%, 39.2%, 12.5%, respectively (Figs 1–2). These findings indicate that the average measurements achieve a higher reproducibility than does just one single measurement.

**Table 3:** The mean difference, paired t-test, 95% LoA and ICC for biometric measurements differences between the two different operators based on a single method (from the first reading from each operator) using OA-2000 swept-source optical coherence tomography in cataract patients.

Device Pairings	Mean Difference ± SD	*P* Value*	95% LoA	ICC (95% CI)
AL (mm)	0.00 ± 0.02	0.845	−0.05 to 0.04	1.000 (0.999 to 1.000)
CCT (μm)	−0.12 ± 4.60	0.825	−9.12 to 8.89	0.990 (0.985 to 0.994)
ACD (mm)	0.00 ± 0.03	0.565	−0.06 to 0.06	0.996 (0.993 to 0.997)
LT (mm)	−0.03 ± 0.15	0.077	−0.33 to 0.27	0.923 (0.882 to 0.951)
Ks (Φ = 2.5) (D)	−0.03 ± 0.18	0.099	−0.40 to 0.33	0.993 (0.989 to 0.995)
Kf (Φ = 2.5) (D)	0.03 ± 0.23	0.209	−0.41 to 0.48	0.990 (0.984 to 0.994)
Km (Φ = 2.5) (D)	−0.00 ± 0.16	0.934	−0.32 to 0.32	0.995 (0.992 to 0.997)
Ks (Φ = 3.0) (D)	−0.01 ± 0.15	0.491	−0.31 to 0.29	0.995 (0.993to 0.997)
Kf (Φ = 3.0) (D)	0.00 ± 0.19	0.820	−0.37 to 0.38	0.993 (0.989 to 0.996)
Km (Φ = 3.0) (D)	0.00 ± 0.13	0.808	−0.26 to 0.25	0.997 (0.995 to 0.998)
WTW (mm)	0.02 ± 0.17	0.262	−0.30 to 0.34	0.897 (0.843 to 0.933)

AL = Axial length, ACD = anterior chamber depth, K = keratometry, WTW = white to white, SD = Standard deviation.

*Two ways.

### Intraoperator repeatability for IOL calculation

Measurements for IOL power calculation performed by both operators are shown in Table 4. Repeatability for IOL power calculation was again excellent for both diameters Ф = 2.5 mm and Ф = 3.0 mm with the ICCs were more than 0.995, the CoVs were less than 0.82%, and repeatability limit (*r*) values less than 0.45D.

**Table 4:** Intraobserver repeatability outcomes for intraocular lens calculation obtained using OA-2000 swept-source optical coherence tomography in cataract patients.

Parameter	observer	Mean ± SD	S_r_	Repeatability limit (*r*)	COV (%)	ICC (95% CI)
SRK/T (Φ = 2.5) (D)	1st	19.78 ± 1.97	0.12	0.33	0.60	0.996 (0.995 to 0.998)
	2nd	19.78 ± 1.98	0.13	0.36	0.67	0.996 (0.994 to 0.997)
Holladay 1 (Φ = 2.5) (D)	1st	19.79 ± 2.04	0.13	0.37	0.68	0.996 (0.994 to 0.997)
	2nd	19.79 ± 2.05	0.15	0.41	0.74	0.995 (0.993 to 0.997)
Hoffer Q (Φ = 2.5) (D)	1st	19.66 ± 2.16	0.15	0.41	0.75	0.995 (0.993 to 0.997)
	2nd	19.66 ± 2.17	0.16	0.45	0.82	0.995 (0.992 to 0.996)
SRK/T (Φ = 3.0) (D)	1st	19.79 ± 1.96	0.11	0.30	0.54	0.997 (0.996 to 0.998)
	2nd	19.80 ± 1.96	0.13	0.36	0.65	0.996 (0.994 to 0.997)
Holladay 1 (Φ = 3.0) (D)	1st	19.8 ± 2.03	0.12	0.33	0.61	0.997 (0.995 to 0.998)
	2nd	19.81 ± 2.03	0.14	0.40	0.72	0.995 (0.993 to 0.997)
Hoffer Q (Φ = 3.0) (D)	1st	19.68 ± 2.16	0.13	0.36	0.67	0.996 (0.995 to 0.998)
	2nd	19.69 ± 2.15	0.16	0.44	0.80	0.995 (0.992 to 0.996)

SD = standard deviation, Sr = within-subject standard deviation, COV = within-subject coefficient of variation, ICC = intraclass correlation coefficient.

### Interoperator reproducibility for IOL calculation

#### Average of 3 consecutive readings

The average of three successive measurements for IOL power calculation performed by both operators are given in Table 5. There was no statistically significant difference between the two operators’ three consecutive mean measurements for IOL power calculation (*P* > 0.05). All the ICCs were more than 0.998, demonstrating excellent reproducibility for IOL power calculation. The 95% LoA was wider when using the 2.5 mm diameter for three formulas. And the SRK/T showed the narrowest 95% LOA both using 2.5 mm and 3.0 mm compared to the other two calculation formulas. Overall, agreement between the two operators was higher when the 3.0 mm, rather than the 2.5 mm, diameter was selected on the OA-2000 to measure K.

**Table 5:** The mean difference, paired t-test, 95% LoA and ICC for intraocular lens calculation differences between the two different operators based on the average method (from average of 3 consecutive readings from each operator) using OA-2000 swept-source optical coherence tomography in cataract patients.

Device Pairings	Mean Difference ± SD	*P* Value*	95% LoA	ICC (95% CI)
SRK/T (Φ = 2.5) (D)	0.00 ± 0.10	0.922	−0.20 to 0.20	0.999 (0.998 to 0.999)
Holladay 1(Φ = 2.5) (D)	0.00 ± 0.12	0.962	−0.23 to 0.23	0.998 (0.997 to 0.999)
Hoffer Q(Φ = 2.5) (D)	0.00 ± 0.13	0.993	−0.25 to 0.25	0.998 (0.997 to 0.999)
SRK/T (Φ = 3.0) (D)	−0.01 ± 0.09	0.461	−0.18 to 0.16	0.999 (0.998 to 0.999)
Holladay 1(Φ = 3.0) (D)	−0.01 ± 0.10	0.501	−0.20 to 0.19	0.999 (0.998 to 0.999)
Hoffer Q(Φ = 3.0) (D)	−0.01 ± 0.11	0.499	−0.22 to 0.20	0.999 (0.998 to 0.999)

SD = Standard deviation, LoA = limits of agreement, ICC = intraclass correlation coefficient.

*Two ways.

#### Single reading

**Figure 3:**
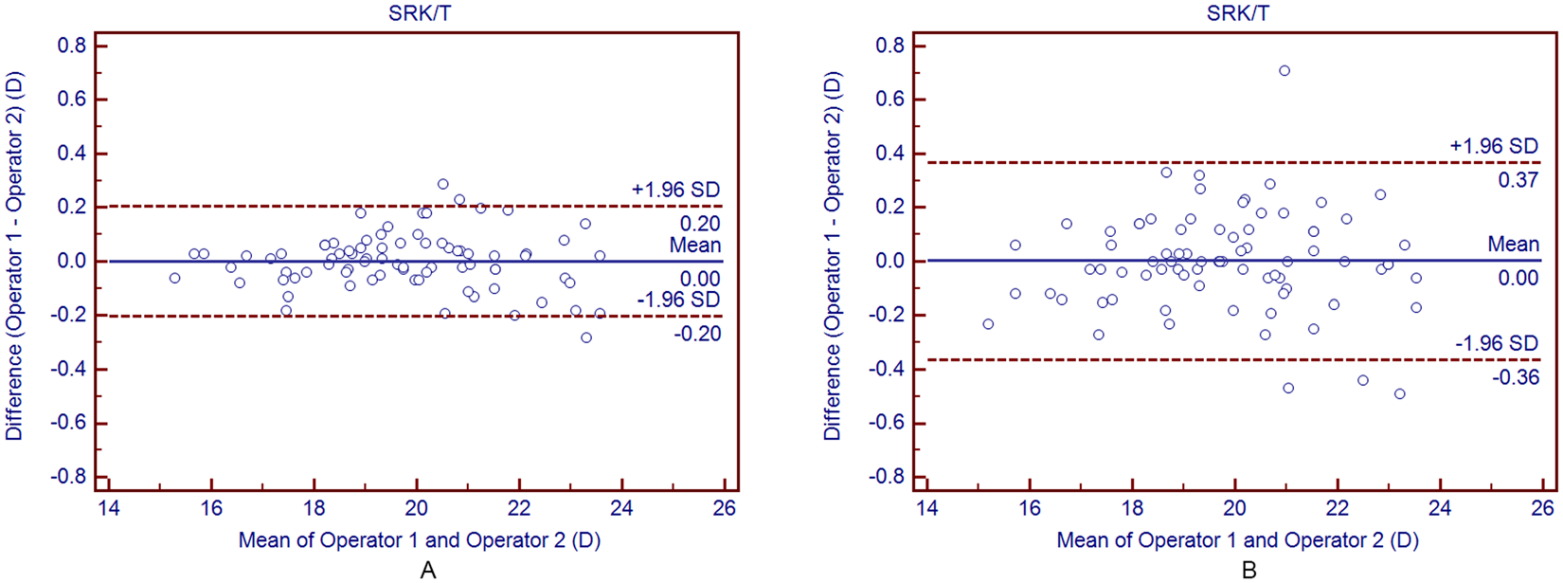
Bland-Altman plots showing agreement in SRK/T formulas calculation between the 2 operators based on average method (**A**) and on the single method (**B**). The solid line indicates the mean difference (bias). The upper and lower dashed lines represent the 95% LoA.

Table 6 shows the interoperator reproducibility of each operators’ first OA-2000 reading for IOL power calculation. All ICCs were over 0.994, revealing excellent reproducibility for three formulas both two diameters. Nevertheless, the 95% LoA were wider than those of three consecutive mean readings (Fig. 3). The width of the 95% LoA that based on the average method using both 2.5 mm and 3.0 mm for SRK/T, Hoffer Q and Holladay I were decreased by 45.2%, 43.9%, 39.3%, 44.3%, 41.8%, 43.2% than those based on the single method, respectively.

**Table 6:** The mean difference, paired t-test, 95% LoA and ICC for intraocular lens calculation differences between the two different operators based on a single method (from the first reading from each operator) using OA-2000 swept-source optical coherence tomography in cataract patients.

Device Pairings	Mean Difference ± SD	*P* Value*	95% LoA	ICC (95% CI)
SRK/T (Φ = 2.5) (D)	0.00 ± 0.19	0.541	−0.36 to 0.37	0.996 (0.993 to 0.997)
Holladay 1(Φ = 2.5) (D)	0.00 ± 0.21	0.092	−0.41 to 0.41	0.995 (0.992 to 0.997)
Hoffer Q(Φ = 2.5) (D)	0.00 ± 0.23	0.565	−0.44 to 0.45	0.994 (0.991 to 0.996)
SRK/T (Φ = 3.0) (D)	0.00 ± 0.15	0.853	−0.30 to 0.31	0.997 (0.995 to 0.998)
Holladay 1(Φ = 3.0) (D)	0.01 ± 0.17	0.706	−0.33 to 0.34	0.996 (0.994 to 0.998)
Hoffer Q(Φ = 3.0) (D)	0.01 ± 0.19	0.924	−0.36 to 0.38	0.996 (0.994 to 0.998)

SD = Standard deviation, LoA = limits of agreement, ICC = intraclass correlation coefficient.

*Two ways.

## Discussion

In the era of refractive cataract surgery, accurate calculation of IOL power requires the most precise and accurate biometric measurements^2,3^. Anatomical parameters, in particular, axial length and keratometry are fundamental to the accurate determination of the most suitable IOL power at the time of cataract surgery. Inaccurate measurements will result in inaccurate IOL power calculations. The OA-2000 is a new optical device based on SS-OCT and Placido disk topography. There is growing evidence of the potential benefits of this new technology (SS-OCT) particularly in terms of acquisition of axial length measurements in dense cataracts^15^. Our recent research showed high reliability on healthy eyes for all ocular parameter measurements using the OA-2000. Especially for AL that showed the highest repeatability (Sw, 0.02 mm, repeatability limit (*r*), 0.07 mm, ICC, 1.0) and reproducibility (ICC, 1.0)^14^. We also found that the refractive outcomes with IOL power calculation based on the measurements by this device were good^16^.

This is the first study to investigate the repeatability and reproducibility of its measurements in patients with cataract. Excellent repeatability and reproducibility were found for all parameters, with AL showing the highest repeatability and reproducibility, and WTW a slightly lower precision. With regards AL, the intraoperator repeatability limit (r) was 0.05 mm and the 95% interoperator LoA ranged from −0.02 to 0.03 mm. Given that in a normal eye, a 0.10 mm error in AL is equivalent to an error of about 0.27D in the spectacle plane^17^, we can state that the errors in the refractive prediction due to AL variability are negligible. The high repeatability and reproducibility of ACD measurements mean that formulas based on this parameter (such as the Haigis, Olsen and Barrett formulas) would not be affected by its variability. Several studies had already evaluated the reproducibility of other optical biometers. Results similar to ours have been reported for other optical biometers, like the IOLMaster^18^, AL-Scan (Nidek Co, Aichi, Japan)^19^, the Aladdin (Topcon Corp, Tokyo, Japan)^20^, and the Lenstar (Haag-Streit, Köniz, Switzerland)^21,22^. K values are one of the main elements for IOL power calculation. The repeatability of Km was slightly better with 3.0 mm readings, but was still very good with 2.5 mm readings too. The repeatability of the OA-2000 K values was similar to that previously reported for other optical biometers, such as the Aladdin^20^ and the Lenstar^22^.

In the present study, the repeatability and reproducibility for WTW and LT measurements were high, but lower than those determined for the other parameters. Similar values have been previously reported for LT with other optical biometers^22,23^. Olsen^24^ highlighted the influence of LT in the prediction of the postoperative IOL position. However, the clinical impact of LT measurement repeatability is still unknown.

WTW is important for phakic IOL implantation. A relatively low repeatability and reproducibility of WTW (similar to ours) have already been reported for the AL-Scan, IOLMaster, Aladdin and Lenstar^20,25,26^. The OA-2000 biometer calculates the WTW by distinguishing the light and shade interface between cornea and sclera. It is likely that, due to the high prevalence of arcus senilis in elderly patients with cataracts, the variations in the method of detection may influence the identification of this edge.

CCT has little influence on IOL power, however, it plays an important role in calculating corrected intraocular pressure, completing any preoperative assessment for keratorefractive surgery and diagnosing corneal diseases, such as Fuch’s corneal dystrophy and keratoconus. OA-2000 measurements of CCT are based on SS-OCT, as is also the case for the IOLMaster 700 (Carl Zeiss Meditec AG). Previous studies with the latter optical biometer found results similar to ours^27–29^.

In the present study, we did not only analyze the reproducibility of the mean measurements obtained by two operators but also analyzed the reproducibility of single measurements. We found that the ICCs for the average parameters were higher and the 95% LoA for the average were narrower than those of a single measurement for both 2.5 mm and 3.0 mm diameters. The ICCs for three IOL power calculation formulas based on the average method were higher and the 95% LoA were narrower than those based on a single method. Our findings were in accordance with our recent studies assessing the corneal power measurement of an RTVue Fourier-domain optical coherence tomography (FD-OCT) system (Optovue Inc., Freemont, CA, USA)^30^. Wang *et al*.^30^ also demonstrated that the width of the 95% LoA was reduced using the mean result rather than the first reading of each operator and recommended the mean for clinical application.

This study has some limitations. First, we only assessed the repeatability and reproducibility of measurements with the OA-2000 in patients with cataracts. The findings cannot be applied to patients with other diseases such as keratoconus or contact lens usage or those who had corneal refractive surgery, so further research is necessary to determine those issues. Moreover, we only investigated the new biometer without comparing its results with those provided by similar instruments based on SS-OCT biometers that have already been validated such as the IOLMaster 700.

In summary, the OA-2000 provides highly repeatable and reproducible estimates of AL, CCT, ACD, K, LT, WTW values in patients with cataracts. For IOL power calculation, we suggest using K values measured at a 3.0 mm diameter. Moreover, because the average values determined from each operator’s 3 consecutive readings were more reproducible, we also recommend that clinicians use the mean values.

## Subjects and Methods

### Patients

Patients with cataracts attending the Eye Hospital of Wenzhou Medical University, Wenzhou, China were invited to participate in this prospective device evaluation study. All patients underwent a complete ophthalmologic examination. Patients with nuclear and cortical score more than 5.0, posterior subcapsular score more than 3.5 based on Lens Opacities Classification System III, previous ocular surgery, trauma, active ocular disease, fundus disease, pre-existing astigmatism >3.0 diopters (D), contact lens usage (within 4 weeks for rigid contact lenses and within 2 weeks for soft contact lenses), poor fixation or significant cognitive impairment were excluded from the study. The study was in compliance with the Declaration of Helsinki. Ethical approval was granted form the ethics committee of the Eye Hospital of Wenzhou Medical University. A written informed consent was obtained from each patient.

### Instrument

The OA-2000 (software version 1.0 R) is a non-invasive, high-resolution, biometry instrument, which combines SS-OCT and Placido disk corneal topography. SS-OCT is a subtype of Fourier domain OCT that can measure AL, ACD, CCT, and LT using a wavelength of 1060 nm. It is equipped with a search function—“B-scanning”, that automatically detects a measurable point when the crystalline lens is unclear. The Placido disk corneal topography is used to measure the radius of corneal curvature based on the Placido disk principle. In addition to the 3.0 mm diameter measured by a general keratometer, a 2.5 mm diameter measurement is simultaneously captured. In this study, the flattest K (Kf) and steepest K (Ks) measurements for each of two areas of differing diameter (Kf (Φ = 2.5), Ks (Φ = 2.5), Kf (Φ = 3.0) and Ks (Φ = 3.0)) were averaged and recorded as mean K (Km) (Φ = 2.5) and Km (Φ = 3.0), respectively. Measurements of the ACD are achieved by means of a charge-coupled device (CCD) camera and infrared light illumination. The WTW distance, i.e. the corneal diameter, was obtained according to the captured image. All measurements with suboptimal quality with poor fixation were deleted and retaken.

### Measurements

Each subject had 3 consecutive measurements, performed by 2 skilled and experienced operators according to the manufactures’ guidelines. For each patient, only the right eye was examined. The initial examiner was assigned randomly in each case. All measurements were performed between 10:00 and 17:00, and all measurements were acquired within a time period of 20 minutes. In this study the following measurements were evaluated: ACD, CCT, LT, WTW, AL, steep K, flat K, and mean K. All patients were positioned correctly in the headrest and asked to fixate on the target without blinking during the scan. Each patient was then instructed to blink completely so as to spread a smooth tear film over the cornea in between scans. When necessary the lids were gently held open (with care not to exert pressure on the globe) to ensure that the lids did not block the corneal mapping area. The IOL power was calculated using the following three formulas: SRK/T (A-constant = 118.0), Holladay 1 (surgeon factor = 1.22), Hoffer Q (predicted ACD = 4.97).

### Statistical Analysis

All data were recorded in a Microsoft Office Excel 2010 (Microsoft Crop, WA, USA) spreadsheet and the statistical analysis was performed using SPSS software for windows V.21 (SPSS Inc. Chicago, Illinois, USA) and MedCalc Statistical Software V16.8 (MedCalc Software, Inc. Belgium). The results of the parameters were expressed by means and standard deviations (SD). The normality of the distribution of data was assessed using the Kolmogorov-Smirnov test (*P* > 0.05). Repeatability (S_r_) equals the within-subject SD (Sw) for repeated measures with the same observer, which is derived by a one-way analysis of variance (ANOVA). The repeatability limit (r) is reported as 1.96√2 × S_r_ which gives the likely limits within which 95% of measurements should occur. S_r_ and r were calculated for the repeated measurements with the three scan modes for each observer^31^. The CoV is defined as the ratio of Sw to the overall mean. A lower CoV is closely related to higher repeatability. The ICC represents the consistency of measurement. The closer the ICC to 1, the better the consistency of measurement is^32^.

To evaluate interoperator reproducibility, the paired t test was used to test the difference of parameters obtained by two operators. Furthermore, agreement between different operators was analyzed with Bland-Altman plots, and the 95% limits of agreement (LOA) were calculated as the mean difference ± 1.96 SDs^33^. A *P* value less than 0.05 was deemed to be statistically significant.
